# Prevalence, duration and risk factors for appendicular osteoarthritis in a UK dog population under primary veterinary care

**DOI:** 10.1038/s41598-018-23940-z

**Published:** 2018-04-04

**Authors:** Katharine L. Anderson, Dan G. O’Neill, David C. Brodbelt, David B. Church, Richard L. Meeson, David Sargan, Jennifer F. Summers, Helen Zulch, Lisa M. Collins

**Affiliations:** 1 University of Lincoln, School of Life Sciences, Lincoln, LN6 7DL UK; 2 Royal Veterinary College, Department of Pathobiology and Population Science, Hatfield, AL9 7TA UK; 3 Royal Veterinary College, Department of Veterinary Clinical Sciences and Services, Hatfield, AL9 7TA UK; 4 University of Cambridge, Department of Veterinary Medicine, Cambridge, CB3 0ES UK; 5 Dogs Trust, London, EC1V 7RQ UK; 6 University of Leeds, Faculty of Biological Sciences, Leeds, LS2 9JT UK

## Abstract

Osteoarthritis is the most common joint disease diagnosed in veterinary medicine and poses considerable challenges to canine welfare. This study aimed to investigate prevalence, duration and risk factors of appendicular osteoarthritis in dogs under primary veterinary care in the UK. The VetCompass^TM^ programme collects clinical data on dogs attending UK primary-care veterinary practices. The study included all VetCompass^TM^ dogs under veterinary care during 2013. Candidate osteoarthritis cases were identified using multiple search strategies. A random subset was manually evaluated against a case definition. Of 455,557 study dogs, 16,437 candidate osteoarthritis cases were identified; 6104 (37%) were manually checked and 4196 (69% of sample) were confirmed as cases. Additional data on demography, clinical signs, duration and management were extracted for confirmed cases. Estimated annual period prevalence (accounting for subsampling) of appendicular osteoarthritis was 2.5% (CI_95_: 2.4–2.5%) equating to around 200,000 UK affected dogs annually. Risk factors associated with osteoarthritis diagnosis included breed (e.g. Labrador, Golden Retriever), being insured, being neutered, of higher bodyweight and being older than eight years. Duration calculation trials suggest osteoarthritis affects 11.4% of affected individuals’ lifespan, providing further evidence for substantial impact of osteoarthritis on canine welfare at the individual and population level.

## Introduction

Osteoarthritis is the most commonly diagnosed joint disease in both human and veterinary medicine^1,2^ Osteoarthritis is typically characterised by progressive degeneration and remodelling of synovial joints, leading to impaired mechanical function of the joint and pain^3^. In humans, osteoarthritis can be an extremely painful and sometimes a debilitating chronic condition that may result from systemic and local biomechanical factors, or develop post-trauma^2^. Apparent gait disturbances and positive response to analgesic therapy in dogs could well reflect similar pain experienced by affected dogs^4^.

Osteoarthritis is usually described as a multifactorial disease with a strong genetic component and can be exacerbated by aspects of lifestyle individual to each dog, including diet and exercise levels^5^. In the dog, osteoarthritis is often described as secondary, whereby a prior primary joint abnormality such as cruciate ligament rupture or patellar luxation is thought to incite the subsequent development of osteoarthritis^3^. Currently, it is unknown what proportion of dogs develop osteoarthritis secondary to these or other specific predisposing conditions^6^.

The prevalence of osteoarthritis in dogs is reported in the literature with conflicting values. Estimates have ranged from 6.6% based on primary-care data^7^ to 20% based on referral data^4^ in the UK dog population. Estimates from North America report age specific prevalence values ranging from 20% in dogs older than one year up to 80% in dogs older than eight years, based on radiographic and clinical data from referral settings^8^.

While it can develop at any age, osteoarthritis is commonly considered a disease of aging and the most commonly reported sites affected by osteoarthritis in the dog include the stifles, hips and elbows^4^. It is generally later in a dog’s life that osteoarthritis becomes a more significant problem and thus the disorder is typically diagnosed when mobility is substantially affected^6^. It is suggested that more than 50% of diagnosed dogs are aged from 8 to 13 years^1^. The length of time that dogs are affected by osteoarthritis is not well reported in the published literature because of difficulty in pinpointing the precise onset of the disease and limited availability of long-term cohort clinical data on confirmed cases. Although osteoarthritis may begin at any age, it may not be clinically diagnosed until it reaches a more advanced stage with obvious external clinical signs^4^. In addition, while joint degeneration may already be present when the initiating cause is diagnosed, it may not yet be recorded or coded as osteoarthritis in the clinical notes at this point. Longitudinal studies have reported that osteoarthritis can affect a substantial proportion of lifespan in some affected dogs^9^.

Several risk factors have been reported for osteoarthritis, suggesting that certain systemic and local factors (animal level factors such as breed, age, sex and obesity) can considerably affect the development of osteoarthritis. Males are frequently discussed as predisposed to osteoarthritis more so than females^10^. This could be due to sex hormone or activity differences as well as differences in bodyweight between males and females^10^. Previous studies have reported that neutered dogs are more likely to develop joint diseases^11^. It is suggested that this association is due to the reduction in gonadal hormones which act as protectors against osteoarthritis^11^, or due to the positive association of neutering with weight gain, whereby higher bodyweight is linked with increasing osteoarthritis development^12^.

Insurance status has been reported to positively affect diagnosis in other joint diseases^13^. For osteoarthritis, this effect is likely due to the cost consideration of diagnostic imaging being removed when dogs are insured and the subsequent increased confidence of diagnosis when supported with radiographic evidence.

Many breed types are reported to be predisposed to developing osteoarthritis, particularly larger breeds, which have higher bodyweights^7^, and purebreds have been considered to be at increased risk of developing osteoarthritis potentially linked to the inherited defects related to conformation of certain breeds^14^.

Finally, the effect of obesity on developing osteoarthritis has been highlighted in previous work. Studies conducted on Labrador retriever litters demonstrated the impact of disease development between dogs fed ad libitum compared with a controlled intake diet, which highlighted that dogs fed ad lib (and therefore more likely to become overweight), were more likely to develop osteoarthritis^9^.

## Study Aims

The aims of this study were (i) to estimate the prevalence of osteoarthritis in the UK dog population; (ii) to identify risk factors for osteoarthritis; (iii) to explore methods of using electronic patient record (EPR) data from veterinary patients, to calculate/reflect the length of time individuals are affected with osteoarthritis, in order to estimate mean durations of osteoarthritis in affected animals and to suggest potentially useful methods for future studies and (iv) to describe how osteoarthritis cases are diagnosed and managed in clinical practice. Aims i and ii will be addressed by testing the following hypotheses: (i) prevalence of osteoarthritis lies between 6% and 20% in the UK dog population; (ii) insured status, older dogs, male dogs, neutered dogs, heavier dogs and purebred dogs are risk groups with increased odds of osteoarthritis diagnosis. Aims (iii) and (iv) will be answered using descriptive analysis of clinical data extracted from manually reviewed EPRs.

## Methods

### Study design

#### Materials

The study used data collected within The VetCompass^TM^ Animal Surveillance programme. VetCompass^TM^ collates de-identified EPR data from primary-care veterinary practices in the UK for epidemiological research^7^. Practices volunteered to participate in the project and to allow extraction of their clinical data from within appropriately configured practice management systems. Information collected included patient demographic (species, breed, date of birth, sex, neuter status, insurance status and bodyweight at various time points) and clinical information. Practitioners could record summary diagnosis terms from an embedded VeNom Code list (a standard set of clinical veterinary terms) during episodes of care. Free-form text clinical notes, treatment and deceased status with relevant dates were also available. EPR data were extracted from practice management systems using integrated clinical queries^15^ and uploaded to a secure VetCompass^TM^ structured query language (SQL) database.

### Availability of materials and data

The datasets generated and analysed during the current study are not publicly available due to their use in ongoing primary research but subsections may be made available from the corresponding author on reasonable request.

### Database search

#### Pilot Study

Initial pilot investigations were conducted to refine a range of search terms used to identify candidate cases within the denominator population. Terms were tested multiple times and refined to accommodate spelling variations and mistakes. A case definition for appendicular osteoarthritis that was applicable to the information available in the EPRs was constructed that aimed to minimise the number of false negative and false positive cases identified. Because many osteoarthritis cases may not undergo diagnostic imaging procedures in primary-care practice, the case definition needed to be broad enough to include common diagnostic protocols typically used in primary-care veterinary practice to diagnose osteoarthritis cases, including combinations of anamnesis, clinical examination, advanced imaging, cytology and other laboratory methods, and trial therapy. A list of data to be extracted on cases to obtain detailed information related to the patient, diagnosis, treatments and behaviour was trialled and created based on availability of data with the EPRs (see Supplementary Note 1 for full list of questions).

An osteoarthritis case was defined as any dog with strong evidence for appendicular skeletal osteoarthritis recorded in the EPR, this included: a final recorded diagnosis or insurance claim for osteoarthritis (or synonym) or that had typical clinical signs and was clinically managed for osteoarthritis e.g. rest, non-steroidal anti-inflammatory drugs (NSAIDs) and supplements (or synonym) or where imaging findings were recorded that were typical of osteoarthritis (e.g. osteophytosis, enthesiopathy, new bone formation, subcondral sclerosis, Morgan’s line).

Exclusion criteria as part of the case definition included:

i. osteoarthritis, degenerative joint disease (DJD) or synonym was only listed as one of a differential list.
ii. an earlier diagnosis of osteoarthritis, DJD or synonym was later revised to exclude osteoarthritis in the clinical notes.
iii. diagnosed only with immune mediated, auto-immune, rheumatoid and septic arthritis/polyarthritis.
iv. osteoarthritis diagnosis related only to non-appendicular locations.

#### Main Study

Candidate case finding used searches conducted on the database to identify potential osteoarthritis cases within a one-year sampling time frame from 1 January 2013 to 31 December 2013. Search terms used were not case-sensitive and covered the clinical notes (osteoa*, OA, degen* + joint*, joint dise*, DJD, osteoph*, arth*) and treatments (cartrophen ~ 2, seraquin, Hill JD, yumove, “mobility treats”, cosequin, Green lipped (mussel), ArthriAid, adequan, specific canine cjd, joint support, chondroitin).

Case confirmation involved manual review of a random subset of EPRs of candidate osteoarthritis cases to discriminate whether the EPR contained sufficient evidence to meet the case definition criteria during 2013. Confirmed cases also included dogs where the records showed continuation of treatment or insurance claims from a pre-existing diagnosis before 2013 (thus including cases both incident (first diagnosed in 2013) and pre-existing (diagnosed pre-2013) in that year). Time constraints precluded extracting detailed data, from the piloted questions, on every case, and therefore every third confirmed case underwent further data extraction to provide a relevant sample size (Supplementary Note 1).

#### Analysis

Relevant records were exported from the VetCompass database to a Microsoft Excel (2016) worksheet for data cleaning and analysis. Descriptive analysis was conducted within Excel for estimation of duration metrics.

The study design used cohort clinical data to classify each animal as either a case or a non-case for osteoarthritis during the study period. Overall, 455,557 dogs were included in the study as a denominator population (Table 1), each qualifying for inclusion by the presence of at least one EPR (clinical note, bodyweight or treatment) either during 2013, or at least one EPR both before and after 2013.

**Table 1:** Population characteristics for the denominator population and candidate population attending primary care practices in the UK.

Population Characteristic		Denominator Population	Candidate Population	Confirmed Cases Population
Number of individuals		455,557	16,437	4196
Median age in 2013		4.1 years; (IQR = 5.9)	9.0 years (IQR = 5.0)	10.5 years (IQR = 5.0)
Sex	Male	234, 212 (51.4%)	8,698 (52.9%)	2,261 (53.9%)
	Female	219,033 (48.1%)	7,715 (46.9%)	1,929 (46.0%)
	Unrecorded:	2,312 (0.5%)	24 (0.1%)	6 (0.1%)
Insurance Status	Insured	31,737 (7.0%)	2,981 (18.1%)	734 (17.5%)
	Uninsured	26,029 (5.7%)	1,225 (7.5%)	335 (8.0%)
	Unrecorded	397,791 (87.3%)	12,232 (74.4%)	3,127 (74.5%)
Neuter Status	Neutered	205,020 (45.0%)	9,405 (57.2%)	2,486 (59.2%)
	Entire	178,218 (39.1%)	3,266 (19.9%)	794 (18.9%)
	Unrecorded	72,319 (15.9%)	3,766 (22.9)	916 (21.8%)
Purebred Status	Purebred	340,769 (74.8%)	12,579 (76.5%)	3,234 (77.1%)
	Crossbred	98,931 (21.7%)	3,568 (21.7%)	953 (22.7%)
	Unrecorded	15,857 (3.5%)	290 (1.8%)	9 (0.2%)

The prevalence estimate was calculated using cross-sectional analysis (accounting for subsampling) from dogs that had osteoarthritis during the study period (2013) and included both incident and pre-existing cases. Prevalence was calculated using the following method: 68.8% of the candidate cases randomly reviewed were confirmed as cases and so it was assumed that 68.8% of all candidate cases were confirmed cases.

Four alternative approaches to reflect duration/temporal length of osteoarthritis effect in individual cases were trialled. Each method made a core assumption that the condition was permanently present following the point of initial diagnosis, even if the condition was successfully clinically managed.

1. Period from date of diagnosis to date of final electronic patient record (for both cases with no recorded death and recorded death)
2. Period from date of diagnosis to date of coding in this study (1/10/2016) (up to date records were available for all cases and therefore those with no recorded death and a final record date before the coding in this study will still have osteoarthritis when coding commenced, as it is incurable)
3. Period from date of first diagnosis to date of death (if death recorded)
4. Percentage of lifespan affected, in cases with recorded death during the study period, i.e. [(date of death - diagnosis date)/age at death].

Median values were calculated for each duration parameter and presented separately for comparison. In addition, the age distribution of cases at diagnosis was determined as a potential indicator of the common age/stage of life at apparent onset of osteoarthritis. Comparison between the age distribution of the denominator population (as of 31^st^ Dec 2013) and age at diagnosis of cases was undertaken. Finally, within Excel, the types of management/treatment of osteoarthritis cases was analysed using descriptive statistics and percentages were calculated for each types of management and treatment methods.

### Statistical Analysis

The age subset data was skewed when tested for normality, therefore a non-parametric Mann-Whitney test was used to test for a statistically significant difference between the median age of the case population (age at diagnosis) against the age of the denominator population (age as of July 1^st^ 2013 for dogs born before this date and the age at December 31^st^ otherwise).

Sample size calculations using Epi Info 7, showed 406 cases and 10,146 non-cases would have 80% power to detect a risk factor with an odds ratio of 1.5 or more (2-sided confidence interval 95%) with a 10% prevalence of osteoarthritis in the unexposed sample. Cross-sectional risk analysis was conducted using SPSS v22 on confirmed cases (both incident and pre-existing) and non-cases (non-cases were defined as all dogs in the denominator population excluding candidates), using both univariable and multivariable binary logistic regression modelling to evaluate the associations of breed, sex, age, Kennel Club (KC) breed group, bodyweight relative to breed, adult bodyweight, insurance, purebred and neuter status with a recorded diagnosis of osteoarthritis. A ‘breed type’ variable included individual breeds with 10 or more osteoarthritis cases, and a general grouping of crossbred as the comparison group. A ‘purebred’ variable categorised all dogs with a recognisable breed name as ‘purebred’, and the remaining dogs as ‘crossbred’. A ‘Kennel Club breed group’ variable categorised breeds grouped according to the relevant UK Kennel Club breed groups (gundog, hound, pastoral, terrier, toy, utility and working). ‘Adult bodyweight’ described the maximum bodyweight recorded during the study period for dogs older than 18 months and was categorised into six groups (0.0–9.9 kg, 10.0–19.9 kg, 20.0–29.9 kg, 30.0–39.9 kg, ≥40.0 kg and unrecorded (including dogs <18 months)). A ‘bodyweight relative to breed’ variable categorised dogs >18 months based on their weight compared to the mean weight calculated for their breed and sex into ‘under the given mean average’, ‘at/over the mean average’ or ‘unrecorded’. Neuter status described the neutering status of the dog (‘neutered’ or ‘entire’) at the final EPR available. Insurance status (‘insured’ or ‘uninsured’) described whether a dog was insured at any point covered by their available EPR. The age variable described the age (years) at the date of first recorded diagnosis of osteoarthritis for confirmed cases. Pre-existing cases were counted as ‘not recorded’. For non-cases this variable described the age on July 1^st^, 2013 for dogs born before this date and the age at December 31^st^ otherwise. Age was entered as a 6-group categorical variable (<3.0 years, 3.0–5.9, 6.0–8.9, 9.0–11.9, ≥12.0, not recorded).

Binary logistic regression modelling was used to evaluate univariable associations between potential risk factors (purebred status, breed type, bodyweight relative to breed, age, sex, KC breed group, neuter and insurance status, adult bodyweight) and a recorded diagnosis of osteoarthritis. Both pre-existing and incident osteoarthritis cases were included. Liberally associated variables (p < 0.20) were carried forward into multivariable modelling. A multivariable model was run excluding purebred and KC breed group (because these were directly co-linear to breed type, as breed type was a main interest variable). Adult bodyweight was also excluded from multivariable model as this is a defining characteristic of individual breeds [method adapted from^16^]. Univariable analysis was used to interpret these variables. Variables included in the multivariable model were: breed type, sex, neuter, insurance status, bodyweight relative to breed mean and age). Model development used an automated backwards elimination (Wald) model in order to remove the least significant variables from the final step model (p < 0.05). Pairwise interactions were tested for all variables in the final multiple model, to explore potential interactions between terms not previously investigated. Biological relevance was considered for pair-wise interactions before putting them into the final model. Statistical significance was set at p < 0.05. The area under the ROC curve was used to evaluate the quality of the model fit.

### Ethics approval

Ethical approval was granted by the College of Science’s Ethics Committee at the University of Lincoln, UK in May 2016 (Reference number CoSREC125).

## Results

### Study Population

The study included 455,557 dogs. Following the initial input of relevant search terms, 16,437 candidate osteoarthritis cases were identified. Of these, 6102 (37.1%) were manually reviewed in detail and 4196 of these candidate cases (68.8%) were confirmed as osteoarthritis cases, with 1259 (30.0% of the 4196 cases) undergoing full data extraction.

### Prevalence estimate

The estimated annual period prevalence of osteoarthritis diagnosis in dogs under primary veterinary care in the UK during 2013 was 2.5% (CI_95_: 2.4–2.5%). Prevalence of the most frequently affected breeds was also calculated with the most prevalent breeds being large breeds, specifically: Golden Retriever (7.7% of all Golden retrievers), Labrador Retriever (6.1% of all Labradors), Rottweiler (5.4% of all Rottweilers) and German Shepherd Dog (4.9% of all German Shepherds) (Table 2).

**Table 2:** Estimated one year period prevalence of osteoarthritis by breed for the most frequent breeds (over 50 individuals) as recorded in primary care practice in the UK during 2013.

Breed	Estimated breed prevalence overall (%)	95% Confidence Intervals (CI)		Number of OA cases sampled	Estimated number of OA cases in population	Number of breed in overall denominator population
		**Lower**	**Upper**			
Golden Retriever	7.74	7.06	8.48	156	421	5439
Labrador	6.13	5.88	6.39	753	2043	33321
Rottweiler	5.43	4.85	6.07	107	289	5321
German Shepherd	4.93	4.56	5.33	224	602	12204
Border Collie	4.52	4.17	4.91	205	554	12264
English Springer	3.36	2.91	3.88	67	181	5384
Springer Spaniel	3.26	2.83	3.75	70	189	5800
Boxer	2.89	2.49	3.32	67	181	6283
Crossbreds	2.60	2.50	2.70	953	2575	98931
West Highland Terrier	2.54	2.27	2.84	113	305	12017
Cavalier King Charles	2.42	2.14	2.74	91	245	10143
Staffordshire Bull terrier	2.22	2.06	2.39	248	673	30275
Yorkshire Terrier	1.23	1.07	1.42	70	189	15426
Cocker Spaniel	0.54	0.48	0.61	95	257	47481
Jack Russel	0.39	0.36	0.45	122	330	82777

### Duration estimate

Median values for proposed osteoarthritis duration indicators/measures were as follows (time in years; interquartile range (IQR); number of dogs contributing to measure):

1. Time from date of diagnosis to date of final record (1.0 years; IQR = 2.0 years; n = 1146)
2. Time from date of diagnosis to date of coding in this study (1.0 years; IQR = 1.0 years; n = 860)
3. Date of first diagnosis to date of death (3.0 years; IQR = 2.0 years; n = 384)

Mean percentage of lifespan affected was 11.4% (CI_95_: 10.0–12.9%), based on 384 cases with recorded date of diagnosis and death.

### Age at Diagnosis

Median age at first diagnosis of osteoarthritis was 10.5 years (IQR = 5.0; lower quartile 6.0, upper quartile 11.0), which differed significantly (p < 0.001) from the median age of the overall denominator population in 2013 at 4.1 years (IQR = 5.9 lower quartile 1.7, upper quartile 7.6).

### Clinical management

Eighty-five percent of osteoarthritis cases were managed with at least one clinical modality (medical or surgical treatments) following osteoarthritis diagnosis. Seventy-five percent of cases were recommended an analgesic, of which 77.9% actually received an analgesic drug. Non-steroidal anti-inflammatory drugs (NSAIDs) were the most frequently used analgesic drug group. Surgical interventions were implemented in 4.8% of cases and 4.6% were referred for further treatment and/or investigations overall. Weight loss was recommended in 25.5% and exercise restriction was recommended in 18.8% of cases. At the final available record, 74.3% of cases remained on medical treatment.

### Risk analysis

Univariable binary logistic regression modelling showed all factors (insurance status, age, sex, bodyweight relative to breed, adult bodyweight, neuter status, purebred status and UK Kennel Club breed group and breed-type) were associated with diagnosis of osteoarthritis (p < 0.20) (Tables 3 and 4) and were subsequently included in various multivariable binary logistic regression model building.

**Table 3:** Univariable logistic regression results for risk factors associated with osteoarthritis diagnosis in dogs attending primary-care veterinary practices in the UK.

Independent Variable	Odds Ratio	95% CI		Significance
		Lower	Upper	
Insurance Status				<0.001
Uninsured	Base			
Insured	1.86	1.63	2.14	<0.001
Age				<0.001
<3 years	Base			
3–5.9 years	4.18	3.40	5.15	<0.001
6–8.9 years	15.75	13.01	19.06	<0.001
9–11.9 years	35.92	29.77	43.36	<0.001
>12 years	58.03	48.11	69.99	<0.001
Sex				<0.001
Female	Base			
Male	1.10	1.03	1.17	0.005
Bodyweight relative to breed mean				<0.001
Under	Base			
At or Over	2.30	2.15	2.45	<0.001
Adult Bodyweight (kg) >18 months				
<10.0	Base			
10.0–19.9	2.84	2.51	3.20	<0.001
20.0–29.9	5.10	4.53	5.73	<0.001
30.0–39.9	8.28	7.37	9.30	<0.001
>40	11.28	10.98	12.76	<0.001
Unrecorded	1.36	1.14	1.62	0.001
Neuter Status				<0.001
Entire	Base			
Neutered	2.68	2.47	2.92	<0.001
Purebred status				<0.001
Crossbreed	Base			
Purebred	1.07	0.99	1.15	0.072
Kennel Club Breed Group				<0.001
Crossbreed	Base			
Gundog	2.28	2.10	2.47	<0.001
Pastoral	2.36	2.13	2.62	<0.001
Hound	0.77	0.62	0.95	0.014
Utility	0.55	0.47	0.64	<0.001
Terrier	1.06	0.95	1.17	0.320
Toy	0.49	0.43	0.57	<0.001
Working	1.49	1.30	1.71	<0.001

**Table 4:** Univariable logistic regression results for breed type associated with osteoarthritis diagnosis in dogs attending primary-care veterinary practices in the UK.

Independent Variable	Odds Ratio	95% CI		Significance
		**Lower**	**Upper**	
Breed Type				<0.001
Crossbreed	Base			
Shih-tzu	0.28	0.20	0.38	<0.001
Labradoodle	0.36	0.20	0.66	0.001
Beagle	0.38	0.22	0.66	0.001
Husky	0.39	0.24	0.64	<0.001
Miniature Schnauzer	0.42	0.26	0.70	0.001
Bichon	0.45	0.31	0.65	<0.001
Jack Russell	0.46	0.38	0.55	<0.001
Yorkshire Terrier	0.46	0.36	0.59	<0.001
British Bulldog	0.49	0.30	0.80	0.004
Lhasa Apso	0.49	0.35	0.70	<0.001
Border Terrier	0.51	0.35	0.74	0.001
Cocker Spaniel	0.61	0.45	0.76	<0.001
Akita	0.74	0.47	1.24	0.256
Lurcher	0.74	0.49	1.12	0.156
King Charles Spaniel	0.81	0.643	1.02	0.073
Staffordshire Bull Terrier	0.82	0.72	0.95	0.006
Basset Hound	0.83	0.44	1.55	0.552
Dogue de Bordeaux	0.83	0.50	1.39	0.483
Bull Mastiff	0.93	0.54	1.62	0.803
West Highland Terrier	0.98	0.81	1.19	0.837
English Bull Terrier	1.01	0.58	1.75	0.981
Weimaraner	1.06	0.65	1.75	0.811
Boxer	1.12	0.87	1.43	0.384
Doberman	1.13	0.70	1.83	0.617
Springer Spaniel	1.30	1.09	1.56	0.004
Hungarian Vizsla	1.35	0.72	2.52	0.354
Greyhound	1.37	0.99	1.88	0.055
Cairn Terrier	1.45	0.94	2.25	0.093
Dalmatian	1.45	0.96	2.17	0.077
Border Collie	1.78	1.53	2.07	<0.001
Shetland Sheepdog	1.83	1.05	3.17	0.032
German Pointer	1.85	1.18	2.89	0.007
German Shepherd	1.98	1.72	2.29	<0.001
Rottweiler	2.17	1.77	2.65	<0.001
Scottish Collie	2.45	1.49	4.04	<0.001
Labrador Retriever	2.47	2.25	2.72	<0.001
Old English Sheepdog	3.05	1.75	5.31	<0.001
Golden Retriever	3.22	2.72	3.81	<0.001

In the univariable analysis for purebred status, purebred dogs were significantly more likely to have a diagnosis of osteoarthritis than crossbreed dogs (p < 0.001, Purebred OR 1.1 CI_95_ 1.0 to 1.2). KC breed type was significantly associated with osteoarthritis diagnosis (p < 0.001,), with the Gundog, Pastoral and Working breed groups all statistically more likely to receive a diagnosis of osteoarthritis compared to crossbreeds (Table 3). Adult bodyweight was statistically significant (p < 0.001) and showed the odds ratios increased with bodyweight with the >40 kg category having the greatest odds of having an osteoarthritis diagnosis (OR 11.3 CI_95_ 11.0 to 12.8).

The final model showed good discrimination (area under the ROC curve: 0.856). In the final multivariable model (Table 5), eleven breeds (Border Collie, Bull Mastiff, Dogue de Bordeaux, German Pointer, German Shepherd Dog, Golden Retriever, Labrador Retriever, Old English Sheepdog, Rottweiler, Scottish Collie and Springer Spaniel) had higher odds of osteoarthritis diagnosis than crossbreeds, with the Rottweiler (OR 3.1, CI_95_ 2.5 to 3.8), Dogue de Bordeaux (OR 2.8, CI_95_ 1.7 to 4.7) and Old English Sheepdog (OR 2.8, CI_95_ 1.6 to 5.0) having the greatest odds ratio. Nine breeds had a statistically decreased association with an osteoarthritis diagnosis compared to crossbreeds. Insured individuals had 2.0 times the odds compared to non-insured dogs (OR CI_95_ 1.8 to 2.3). Neutered individuals had 1.8 (CI_95_ 1.7 to 2.0) times the odds of diagnosis compared with entire dogs. Males had an odds ratio of 1.2 (OR CI_95_ 1.1 to 1.3) compared to females. The odds of osteoarthritis increased with increasing age (p < 0.001). Bodyweight relative to breed mean was also significant in the final model (p < 0.001), with the model indicating that dogs that were at or over average weight had increased odds ratio for having a diagnosis of osteoarthritis compared to dogs that were under the average weight for their breed and sex (OR 2.3, CI_95_ 2.1–2.4).

**Table 5:** Results of multivariable binary logistic regression for risk factors associated with diagnosis of osteoarthritis in dogs attending primary-care practices in the UK.

Independent Variable	Numbers in population		Odds Ratio	95% CI		Significance
	**Case**	**Non-case**		**Lower**	**Upper**	
Insurance Status						<0.001
Uninsured	304	20,745	Base			
Insured	651	23,801	2.02	1.76	2.33	<0.001
Age						<0.001
<3 years	120	136,217	Base			
3 to 5.9 years	348	94,496	3.55	2.88	4.37	<0.001
6 to 8.9 years	872	62,864	12.58	10.37	15.25	<0.001
9 to 11.9 years	1191	37,634	28.83	23.84	34.86	<0.001
>12 years	1306	25,548	53.89	44.57	65.15	<0.001
Sex						<0.001
Female	1772	174,167	Base			
Male	2068	185,628	1.19	1.11	1.27	<0.001
Neuter Status						<0.001
Entire	717	139,212	Base			
Neutered	2303	166,682	1.80	1.65	2.00	<0.001
Breed						<0.001
Crossbreed	953	95,363	Base			
Rottweiler	107	4940	3.11	2.53	3.83	<0.001
Old English Sheepdog	13	427	2.81	1.59	4.98	<0.001
Dogue de Bordeaux	15	1803	2.81	1.67	4.73	<0.001
Labrador Retriever	758	30,679	2.56	2.31	2.82	<0.001
Golden Retriever	163	5071	2.42	2.03	2.88	<0.001
German Shepherd	231	11,656	2.28	1.96	2.64	<0.001
Bull Mastiff	13	1395	1.82	1.04	3.17	0.035
Scottish Collie	16	654	1.77	1.06	2.94	0.029
German Pointer	20	1081	1.62	1.03	2.55	0.038
Border Collie	205	11,551	1.51	1.29	1.76	<0.001
Springer Spaniel	138	10,616	1.25	1.05	1.51	0.015
Cocker Spaniel	95	15,477	0.70	0.57	0.87	0.001
West Highland Terrier	113	11,543	0.66	0.54	0.81	<0.001
Bichon	29	6484	0.62	0.42	0.89	0.011
Lhasa Apso	33	6720	0.58	0.41	0.83	0.003
Miniature Schnauzer	16	3780	0.55	0.33	0.90	0.017
Border Terrier	27	5334	0.53	0.36	0.78	0.001
Shih-tzu	41	14,873	0.47	0.34	0.64	<0.001
Jack Russell	124	27,119	0.41	0.34	0.50	<0.001
Yorkshire Terrier	70	15,114	0.40	0.31	0.51	<0.001
Bodyweight relative to breed average mean						<0.001
Under	1426	178,081	Base			<0.001
At or Over	2535	137,748	2.29	2.14	2.44	

## Discussion

In this study, annual period prevalence was calculated at 2.5% from a population of 455,557 dogs attending primary-care practices. This prevalence estimate of 2.5% is significantly lower than the previous estimates^7^ and^4^ which ranged from 6.6%^7^ to 20%^8^. Whilst the 6.6% estimate was also primary-care data, the dataset was smaller than this current study (sample population of 148,741), the time frame was much greater and that study did not use as tight a case definition as this study^7^. Other prevalence estimates have been suggested to be as high as 20% prevalence in dogs over one year of age, however this is based on the North American referral dog population from 1997^8^. The methodology of the North American study used radiographic diagnoses as cases, as structural changes due to osteoarthritis can be confirmed^8^. However, another study showed that radiographic evidence of osteoarthritis correlates poorly with clinical limb function of dogs with osteoarthritis, which highlights a limitation of a radiographic only diagnosis study^17^. The use of primary-care data has been suggested to be more representative of the general dog population, as referral populations are sub-selected based on the severity and complexity of the cases and inherently lead to bias^18^. The majority of referral populations tend to have at least one illness to justify referral, whereas many animals in primary-care populations have no illnesses and instead present for routine or prophylactic reasons^7^. This may have the effect of making referral populations have apparently higher prevalence values compared with primary-care populations, especially for severe or complicated disorders that are more likely to get referred^19^.

It should be noted that our study may give a conservative prevalence estimate due to the nature of data acquisition. A tight case definition was designed to increase the specificity of the study; however, some true cases may have been excluded due to insufficient information available in the EPR. Additionally, primary-care data relies heavily on the input from the veterinarian treating the individual, resulting in differing examination, diagnostic and note-taking preferences. Some true osteoarthritis cases may not have been included due to lack of follow up or because osteoarthritis was listed only as one of a list of differential diagnoses, along with other primary joint-diseases, such as dysplasia. Irrespective of likely under-estimation, based on an estimated UK dog population of 8.5 million dogs (2017 Pet Food Manufacturers’ Association), the prevalence figure suggests at least 200,000 dogs are affected by osteoarthritis annually in the UK and therefore still provides substantial evidence for major welfare impact at a UK population level. In terms of welfare, the actual prevalence of ‘joint disease’ will be significantly higher, than this osteoarthritis prevalence would imply.

Four calculation methods were trialled in this study as exploratory methods to estimate disease duration. The most definitive of these calculations were the date of first diagnosis to date of death and the subsequent calculation of percentage of life span from these same numbers. These are the safest data as they give the latest value of the true start and end dates; however, the data were limited to only the dogs that died during the case of the study which, in the case of this study, was 384. The other two calculations were limited by data availability but had larger sample sizes; however, these do not reflect the duration as well, as they use estimated end dates or incomplete end dates and so the figures may be inaccurate and duration may actually be much greater when death is actually reached. In order to report duration more accurately, duration studies with full cohort analysis using reliable date points from thousands of individuals (birth date-diagnosis date-death date) would provide the most accurate representation of disease duration in diseases that are permanent. However, where this is not available, date of diagnosis to date of death provides the most valuable tool for assessing duration, even if the sample is relatively small. In the current study, the percentage of life affected by osteoarthritis was reported as 11% based on the sub-set of cases with recorded death in available EPRs. Many osteoarthritis cases may well be diagnosed later in life, but earlier signs may be missed or still considered normal to the owner and may only become apparent when the animal ages or when debility reaches more noticeable levels. Therefore, many more dogs could be suffering for a longer duration than what is recorded in EPRs, with implications for welfare^20^.

Crossbreeds are often considered to have health benefits from hybrid vigour because of reduced homozygosity^21^. In this study, purebreds showed slightly increased odds ratio (1.1) for osteoarthritis compared to crossbreeds, with the increased risk of developing osteoarthritis potentially linked to the inherited defects related to conformation in particular breeds^14^. However, other studies have also shown that crossbreeds may be more prone than purebreds to joint disorders such as cruciate ligament rupture, that can lead to osteoarthritis development^22^. This increase in crossbreeds, could be due to individuals that genetically aren’t suitable for purebred breeding, are used in breeding crossbreeds. It may be the case that the parent breeds of crossbreds include at risk breeds (e.g. larger breeds or Labradors), and a genetically predisposed crossbred may also result, increasing their susceptibility to disorders such as osteoarthritis, instead of creating the desired hybrid vigour that crossbreeding is often claimed to achieve^21^.

Eleven breeds had significantly higher diagnosis of osteoarthritis compared to crossbreed dogs in the current study. These included: Border Collie, Bull Mastiff, Dogue de Bordeaux, German Pointer, German Shepherd Dog, Golden Retriever, Labrador Retriever, Old English Sheepdog, Rottweiler, Scottish Collie and Springer Spaniel. These findings are consistent with previous studies^7,23^ and^24^. These are all medium/large breeds and therefore have high bodyweight. All of these breeds fall under the working, pastoral and gundog KC breed types, which were also found to have increased odds of developing OA. This could be similar to occupational osteoarthritis seen in humans as a result of increased physical workload on the joints^25^. The classification of a dog as being of, for example, a ‘working’ breed group may however, bear very little relationship with how much ‘work’ or exercise the dog actually gets. True levels of ‘work’ or exercise that the dogs in this study received was not available, and therefore further studies to investigate this potential comparison with occupational osteoarthritis in humans would be beneficial. Many smaller breeds in the current analysis (e.g. Yorkshire Terrier, West Highland Terrier and Shih-tzu) had statistically significantly reduced odds of an osteoarthritis diagnosis compared to crossbreeds. Smaller breeds may show less obvious lameness/apparent pain than larger breeds and therefore receive lower diagnostic proportions than the breeds, with studies showing that lameness assessments are difficult due to gait differences^26^.

Previous research suggested that male dogs have greater odds of developing osteoarthritis than females^10^, and that neutered dogs have higher odds of developing cruciate ligament problems and hip and elbow dysplasia, with associated higher risk of secondary osteoarthritis^13,27^ and^28^. In the current study, males had 1.2 times the odds of osteoarthritis and neutered individuals had 1.8 times the odds. Neutering may reduce gonadal hormone protection of joints compared with entire individuals^13,27,28^. An association between neutering and weight gain has also been previously demonstrated^29^ and could explain part of the increase in neutered dogs. Neutering has also been shown to affect the satiety of individuals due to changes in hormones such as oestrogen^29^. Adult bodyweight was identified as a risk factor, with increasing odds of having an osteoarthritis diagnosis with increasing bodyweight, suggesting that dogs that are neutered and subsequently gain weight, may be more likely to develop osteoarthritis. It should be noted however, without also having morphometric data, (e.g. body condition), it is difficult to know whether bodyweight reflects body condition (e.g. obesity) or just simply stature/size. It could also be the case that dogs below breed average weight describe a smaller framed subset of the breed and that this may influence the risk of the development of osteoarthritis. This would be a valuable factor to follow up, especially in breeds where breed standards tend to favour larger individuals. It is also conceivable that increased weight gain could be secondary to the osteoarthritis development due to reduced energy expenditure when debilitated by osteoarthritis or when physical activity is reduced, and hence neuter status could have a different mode of influence. In individuals that truly are obese, the mechanism increasing the likelihood of developing osteoarthritis could be related to the levels of leptin (shown to be higher in overweight individuals) which have been shown to have a damaging effect on articular cartilage and thus increase the risk of osteoarthritis^12^.

The results from the current study indicated that age of diagnosis was most frequently eight years and over and study dogs over twelve years had the highest odds of osteoarthritis diagnosis compared to other age groups, supporting studies that suggest osteoarthritis is a disease of aging^30^, similar effects have also been shown in humans^2^. While this finding could suggest that osteoarthritis is occurring more in older dogs and the associated morbidity is strongly related to increasing age, it may also indicate that osteoarthritis is generally only noticed and/or investigated to the stage of formal diagnosis later in life. In addition, osteoarthritis can present differently symptomatically, for example from a seemingly unnoticeable altered gait or behaviour to severe limping or lameness^4^ and therefore may not necessarily be picked up by the owner until it has progressed to a certain severity^4^. It has also been shown that in many cases owners don’t often recognise problems or changes in their dog related to inherited defects, such as osteoarthritis. Due to normalisation of certain health traits, some owners do not perceive changes, for example altered gait, as problems, but consider them normal^20^ and therefore presentation of osteoarthritis cases for veterinary attention may be delayed until changes worsen and become more apparent to the owner.

Insurance status was a significant ‘risk factor’ for diagnosis with osteoarthritis, with insured individuals demonstrating twice the odds of osteoarthritis diagnosis compared with non-insured individuals. A similar positive association for diagnosis has also been reported for other diseases^13^. In the instance of osteoarthritis, diagnostic imaging methods for diagnosis confirmation as well as the long-term nature of the condition and therefore treatment and costs are likely to explain much of the increased diagnosis associated with insurance. It may be the case that uninsured individuals are more likely to receive an uncertain diagnosis (i.e. the diagnosis was part of a differential diagnosis) and/or no follow up diagnosis in the EPRs due to the owner not presenting their dog to the vet for follow-up, meaning they are excluded as cases from the study according to the inclusion criteria. This scenario could suggest compromised welfare in these uninsured, osteoarthritis-affected dogs, through lack of appropriate treatment or monitoring.

In relation to clinical management of osteoarthritis, 85.1% of cases were managed with at least one treatment following diagnosis, 75.7% of which included the use of an analgesic drug. This highlights the 24.3% of cases that were not administered analgesics. It is impossible to determine if this relates to a veterinarian^31^, or owner preference, or indeed disease severity, however it may suggest a welfare concern for these dogs. Surgery was used in 4.8% of cases, and 4.6% were referred for further investigation and/or treatments. The low surgical intervention following the diagnosis of osteoarthritis, may be due to the costs or risks relating to the surgery (particularly older dogs), joint unsuitability, or resulting implications for quality of life that can occur during rehabilitation and recovery, or simply due to the fact that surgery is not appropriate for many cases. Eighty percent of cases in this study were managed using an analgesic treatment, prescribed at the primary-care consultations, 97% of these were NSAIDs. Whilst continual long-term use of NSAIDs has been suggested to sufficiently alleviate osteoarthritis pain, protracted use can lead to complications and impact quality of life, through the development of gastrointestinal problems, or organ damage, such as kidney or liver^32^, as well as long term costs and thus owners may be wary of continual use.

Exercise restriction (defined as any recommendation to reduce or stop exercise for any duration) was recommended in 21% of osteoarthritis cases. Osteoarthritis can significantly affect the gait of a dog, which in turn can impact unaffected joints and therefore increase the negative impact of the disease for that individual^33^. Exercise restriction can play an important role, preventing further damage to other joints until pain can be managed, for example by dietary management or analgesic methods^33^. Exercise is recommended to be restricted to small and frequent exercise but not stopped totally as this can be detrimental to the general health of the dog and joints themselves^4^. For an active species, however, exercise can be extremely important both physically and mentally^34^, so this finding suggests canine welfare sometimes is affected short-term through management (e.g. where osteoarthritis affects the biological need for exercise and activity as part of behaviour) in order to improve the welfare in the long-term.

Twenty-eight percent of cases were recommended to undergo weight loss. This demonstrates awareness that weight loss is beneficial for osteoarthritis^35^. The current study reports that dogs at or above mean breed bodyweight were 2.3 times more likely to have osteoarthritis than dogs below average weight. Therefore, this recommendation to undergo weight loss may support a correlation between excessive bodyweight/obesity and the development or severity of osteoarthritis. However, it could also suggest cases diagnosed with osteoarthritis may go on to gain weight, perhaps through reduced exercise. Reducing food intake may again reduce the quality of life in the dog, a species that is well known for being food motivated^36^. It has been shown that diet alterations and food restrictions can have an effect on the behaviour and wellbeing of dogs^37^, demonstrating further that management of osteoarthritis can have a large impact on canine welfare and specific interventions must be carefully considered to ensure they outweigh the implications of not implementing them.

Whilst there is great potential from analyses of large datasets such as this to better understand the welfare impact of a condition, the study had a number of limitations. Firstly, only dogs who were under veterinary care were included in this study and consequently the results may not be fully generalisable to the entire wider population, some of which may rarely attend a veterinary surgery, or not be registered at all and thus underrepresented in this study. Although this study included a convenience sample of both independent and linked primary-care practices that extended across England, Wales, Scotland and Northern Ireland, the majority were linked within larger practice groups and therefore may not be fully representative of the overall practices in the UK. The data are only as reliable as the notes recorded by the veterinary teams and the information provided by the owners. This study required the veterinarians to make the diagnoses of osteoarthritis, as well as record appropriate data in the EPRs. It should be noted that there is no definitive way of knowing the date when osteoarthritis first developed, or progressed to osteoarthritis from a predisposed disease process. Therefore, there are possibilities that some dogs with osteoarthritis were not diagnosed at all or that some recorded were also misdiagnosed. However, overall, all Royal College of Veterinary Surgeons-registered veterinarians should be well-placed to make a clinical diagnosis of osteoarthritis in primary-care practice and hence this study is likely to present an accurate overall representation. The denominator population included all dogs that were under veterinary care at a VetCompass^TM^ practice in 2013, whether presenting healthy (for preventative medicine consultations) or sick (for any reason).

## Conclusion

This study reports an annual period prevalence estimate of 2.5% for appendicular osteoarthritis in the UK dog population. Based on UK dog population estimates of 8 million this equates to just over 200,000 individuals affected with osteoarthritis in a given year. It was also shown that diagnosis was usually made when dogs were older. In cases with available dates of diagnosis and death mean proportion of lifespan affected was 11%. Risk factors for osteoarthritis showed a diagnosis was more likely in older individuals, males, neutered dogs, in dogs with higher bodyweights and insured dogs. Nine statistically predisposed breeds were highlighted by the analysis, with Old English Sheepdogs, Rottweilers and Dogue de Bordeauxs having the highest odd ratios. Clinical management of osteoarthritis was implemented in 90% of cases with use of analgesics being the most frequently used therapeutic intervention.

Taken together, findings of this study suggest that osteoarthritis poses a notable challenge to canine welfare with respect to numbers of dogs affected and perceived requirement for veterinary therapy including frequent use of analgesia. The suggested breed predispositions, duration of effect and the scale of welfare impact at the UK population level would benefit from investigation in greater depth.

## Electronic supplementary material

Supplementary Information
