# Supplementary material for: Prevalence, duration and risk factors for appendicular osteoarthritis in a UK dog population under primary veterinary care
Source: Sci Rep. 2018 Apr 4;8:5641. doi: 10.1038/s41598-018-23940-z (PMC5884849; doi:10.1038/s41598-018-23940-z)
Supplement: Supplementary file 1 — Supplementary Information [file 41598_2018_23940_MOESM1_ESM.pdf]

Prevalence, duration and risk factors for appendicular osteoarthritis in a UK dog population under primary veterinary care

Katharine L. Anderson, Dan G. O'Neill, David C. Brodbelt, David B. Church, Richard Meeson, David Sargan, Jennifer F. Summers, Helen Zulch, Lisa M. Collins

Supplementary Note 1: Questions asked of the EPR data as part of data extraction

---

<u>Patients:</u>

Is the dog an OA case?

<u>Problem Findings:</u>

Was osteoarthritis (or synonym) specifically recorded as a final diagnosis?

Incident or pre-existing in 2013?

Date of first diagnosis at any time in the clinical records

<u>Diagnosis</u>

Was the dog radiographed ± 3 months from the diagnostic episode of care?

Was there presence of osteophytes?

Did the dog have a CT ± 3 months from the diagnostic episode of care?

Single vs. multiple joints affected ± 3 months from the diagnostic episode of care?

<u>Presenting signs and symptoms</u>

Was thickening of the joint (or synonym) recorded ± 3 months from the diagnostic episode of care?

Was the presence of crepitus (or synonym) recorded ± 3 months from the diagnostic episode of care?

Was limping/lameness recorded ± 3 months from the diagnostic episode of care? Was stiffness/reduced range of movement (or synonym) in the effected joint/s recorded ± 3 months from the diagnostic episode of care?

Behaviour

Did the owner report changes in normal behaviours/addition of new behaviours ± 3 months from the diagnostic episode of care?

(Alterations in behaviour include soiling the house, panting, fidgeting, or generalised 'behaviour change')

Did the owner report that the dog has become aggressive at home or out of the home (to people or other dogs) ± 3 months from the diagnostic episode of care?

Did the owner report the onset of fears (for example noise fear) ± 3 months from the diagnostic episode of care?

Did the owner report that the dog is reluctant to exercise ± 3 months from the diagnostic episode of care?

Did the owner report that the dog is moody ± 3 months from the diagnostic episode of care?

Did the owner report that the dog is quieter than before ± 3 months from the diagnostic episode of care?

(Quieter defined as less vocal, lethargic, slower or lack of appetite/thirst Did the owner report any increase in vocalisations than before ± 3 months from the diagnostic episode of care?

(Vocalisations defined as howling, whining. Whimpering, yelping and barking) Did the owner report that the dog is calmer than before ± 3 months from the diagnostic episode of care?

(Calmer defined as less playful, sleeping more or tires more easily)

Treatment

Was at least one veterinary treatment recommended for osteoarthritis at any time in the clinical records?

Was at least one veterinary treatment prescribed/administrated for osteoarthritis at any time in the clinical records?

(Veterinary treatment was defined as any pain relief, diet/weight management, supplement nutraceutical, hydro/physiotherapy, laser therapy or acupuncture used to manage the OA)

Was at least one analgesic agent recommended for osteoarthritis at any time in the clinical records?

Was at least one analgesic agent prescribed/administered for osteoarthritis at any time in the clinical records?

(Analgesia was defined as any corticosteroids, NSAIDS or Opioids)

What type of Analgesia type prescribed? (Glucocorticoid, NSAID, Opioid, or combination of these)

Was the analgesia prescribing/administration ± 3 months from the diagnostic episode of care??

Was there use of structure-modifying treatments for OA at any time in the clinical records? (E.g. cartrophen?)

Was weight loss/management recommended?

Was exercise restriction recommended?

Was surgical intervention/procedure recommended?

Was surgical intervention/procedure performed?

Was referral recommended for investigation/therapy?

Was referral undertaken for investigation/therapy?

Was the dog on treatment for OA at the final record of OA?

Final Record information

Date of final record overall

Single vs multiple joints affected at end of record for OA?

Did the dog die during the study?

Date of death

Method of death (euth/unassisted/unrecorded)

Did OA contribute to the death?
